# *ERBB2* mutation is associated with a worse prognosis in patients with *CDH1* altered invasive lobular cancer of the breast

**DOI:** 10.18632/oncotarget.13019

**Published:** 2016-11-02

**Authors:** Zheng Ping, Gene P. Siegal, Shuko Harada, Isam-Eldin Eltoum, Mariam Youssef, Tiansheng Shen, Jianbo He, Yingjie Huang, Dongquan Chen, Yiping Li, Kirby I Bland, Helena R Chang, Dejun Shen

**Affiliations:** ^1^ Division of Anatomic Pathology, University of Alabama at Birmingham, Birmingham, AL, USA; ^2^ Division of Cell and Molecular Pathology, Departments of Pathology, University of Alabama at Birmingham, Birmingham, AL, USA; ^3^ Division of Hematology/Oncology, Department of Medicine, University of Alabama at Birmingham, Birmingham, AL, USA; ^4^ Division of Preventive Medicine, Department of Medicine, University of Alabama at Birmingham, Birmingham, AL, USA; ^5^ Division of Surgical Oncology, Department of Surgery, University of Alabama at Birmingham, Birmingham, AL, USA; ^6^ UAB Comprehensive Cancer Center, University of Alabama at Birmingham, Birmingham, AL, USA; ^7^ Ningbo Clinical and Pathological Diagnosis Center, Ningbo, Zhejiang, China; ^8^ Revlon/UCLA Breast Center, Department of Surgery, University of California at Los Angeles, David Geffen School of Medicine, Los Angeles, CA, USA

**Keywords:** invasive lobular carcinoma, CDH1 mutation, ERBB2 mutation, genomics, TCGA

## Abstract

E-cadherin (*CDH1*) is a glycoprotein that mediates adhesion between epithelial cells and also suppresses cancer invasion. Mutation or deletion of the *CDH1* gene has been reported in 30–60% cases of invasive lobular carcinoma (ILC). However, little is known about genomic differences between ILC with and without a *CDH1* alteration. Therefore, we analyzed whole genome sequencing data of 169 ILC cases from The Cancer Genome Atlas (TCGA) to address this deficiency. Our study shows that *CDH1* gene was altered in 59.2% (100/169) of ILC. No significant difference was identified between *CDH1*-altered and -unaltered ILC cases for any of the examined demographic, clinical or pathologic characteristics, including histologic grade, tumor stage, lymph node metastases, or ER/PR/HER2 states. Seven recurrent mutations (*PTEN*, *MUC16*, *ERBB2*, *FAT4*, *PCDHGA2*, *HERC1* and *FLNC*) and four chromosomal changes with recurrent copy number variation (CNV) (11q13, 17q12-21, 8p11 and 8q11) were found in ILC, which correlated with a positive or negative *CDH1* alteration status, respectively. The prevalence of the most common breast cancer driver abnormalities including *TP53* and *PIK3CA* mutations and *MYC* and *ERBB2* amplifications showed no difference between the two groups. However, *CDH1*-altered ILC with an *ERBB2* mutation shows a significantly worse prognosis compared to its counterparts without such a mutation. Our study suggests that *CDH1*-altered ILC patients with *ERBB2* mutations may represent an actionable group of patients who could benefit from targeted breast cancer therapy.

## INTRODUCTION

The most recent WHO Classification of Tumors of the Breast (2012) has described over 20 special types of invasive breast cancer [1]. Among them, invasive breast cancer “of no special type” or invasive ductal carcinoma (IDC) consists of the majority of cases (60–70%), and invasive lobular carcinoma (ILC) is the most common special subtype, representing 5–15% of all invasive breast cancers. ILC is a histologic diagnosis, and by definition, it composes of non-cohesive tumor cells individually dispersed or arranged in a single-file linear pattern in a fibrous stroma [1]. The ILC tumor cells are typically estrogen receptor (ER) positive and epidermal growth factor receptor 2 (HER2) negative, and often have a lost or aberrant epithelial cadherin (E-cadherin, *CDH1*) protein expression. While most ILC belongs to so-called classic variant consisting of small, monotonous and non-cohesive tumor cells individually dispersed or arranged in a single-file linear pattern, multiple ILC histologic variants have also been described, including solid, alveolar, and trabecular variants based on their architectural patterns, or pleomorphic, apocrine, histiocytoid, and signet ring cell variants based on their cytology [2, 3]. Although the accumulating outcome data on different ILC variants are sometimes conflicting, there has been a recent consensus that the histologic grading features including nuclear pleomorphism and mitotic count are probably the most useful independent prognostic factors [1, 2, 4].

E-cadherin is a cell-cell adhesion molecule that plays an important role during development and carcinogenesis [5]. Loss of E-cadherin is the hallmark of ILC and has been used to confirm the diagnosis of such neoplasms clinically when it is necessary [1, 2]. Although loss of E-cadherin is predominantly seen in lobular neoplasms, a range of approximately 10% up to 30% of ILC are positive for E-cadherin as measured by immunohistochemical staining [2, 6]. In contrast, while most ductal carcinomas are strongly positive for E-cadherin, a small portion of IDC are seen to be E-cadherin negative [7, 8]. Although mutation or deletion of *CDH1* gene has been well recognized to be responsible for loss of E-cadherin protein expression in lobular neoplasms, molecular mechanisms underlying ILC with and without a *CDH1* alteration are not well studied. The data from the COSMIC database shows that only 35% of ILC (83/235) carry a *CDH1* mutation, while three recent, large scale genome sequencing studies have shown that 43% to 65% of the ILC had a *CDH1* gene mutation or deletion [9–11]. The study based on TCGA breast cancer sequencing data had also demonstrated that most of the remaining ILC without a *CDH1* mutation also harbor various E-cadherin gene abnormalities in their transcriptional and/or translational pathways [9]. However, it remained unknown as to whether there were any differences in clinical, pathological and genetic characteristics between ILC with and without a *CDH1* alteration.

Using the genomic sequencing data from TCGA Breast Invasive Carcinoma Cohort, we have recently published a unique microscopic landscape of IDC [12]. We also previously reported that ILC generally has fewer genomic abnormalities including a lower burden of gene mutations and copy number variations (CNV) compared to IDC [13]. In this study, we have further demonstrated that ILC with and without *CDH1* alterations are strikingly similar clinically, pathologically, and even genetically. However, an *ERBB2* mutation was co-occurred with *CDH1* mutation and associated with a worse prognosis. This finding may shed light on designing precision or targeted therapy for ILC.

## RESULTS

### Clinical and pathological characteristics of TCGA ILC cohort with and without a *CDH1* alteration

**Figure 1:**
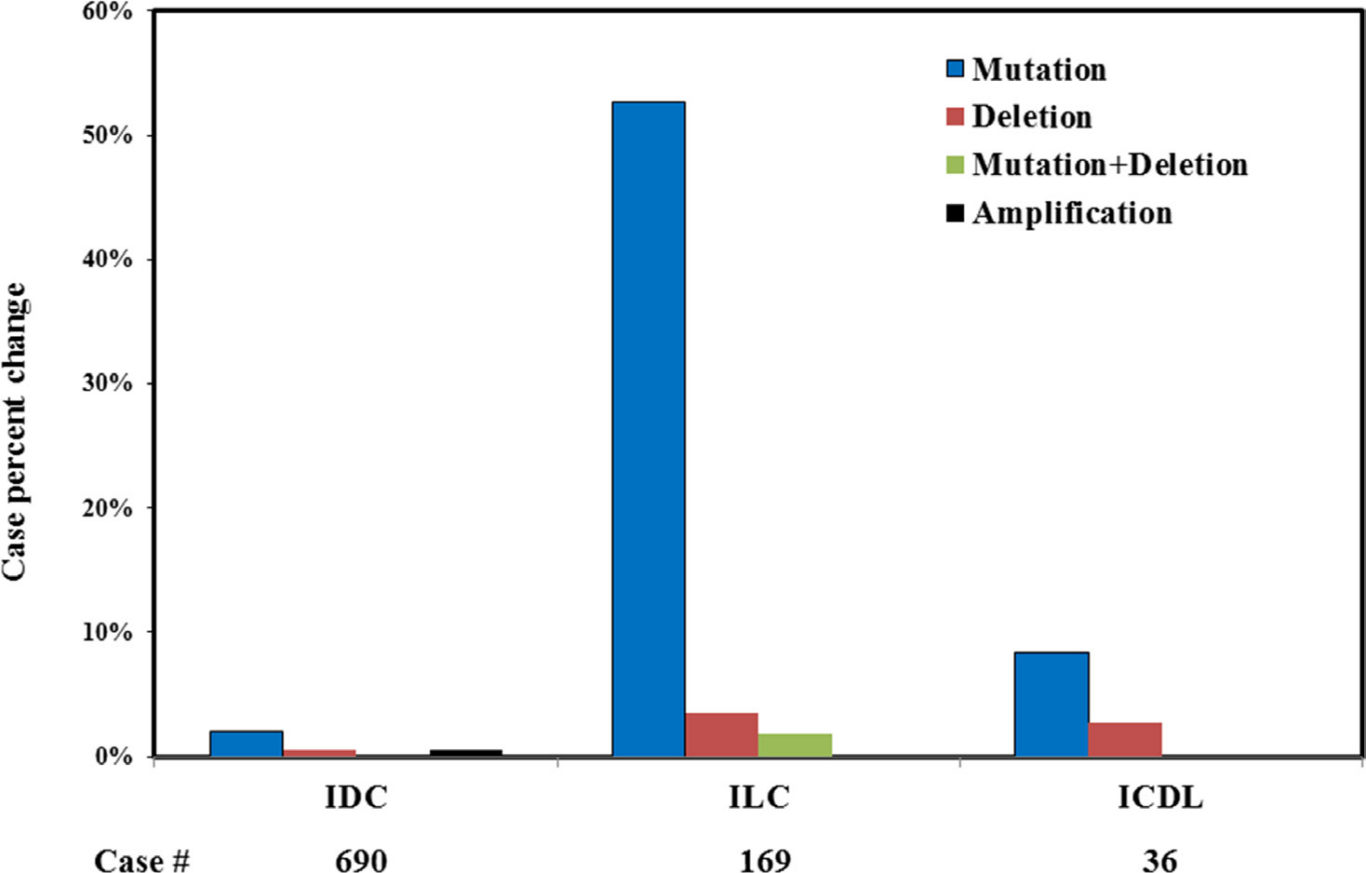
*CDH1* genetic abnormalities in different types of invasive breast cancer The data is from Breast Invasive Carcinoma, TCGA provisional.

The Cancer Genome Atlas (TCGA, http://cancergenome.nih.gov/) is an international database collecting and hosting large scale genomic sequencing data across different types of cancer. The Breast Invasive Carcinoma (TCGA, provisional) Project, as of July 1, 2016, contains 690 cases of IDC, 169 cases of ILC, 36 cases ICDL (invasive carcinoma with ductal and lobular features), and 210 cases of other subtypes of invasive breast cancer. *CDH1* was the most frequently mutated gene in the TCGA ILC cohort, which caused a truncated protein (90%) and was identified in 53.8% (91/169) of the ILC cases, while another nine ILC cases had a *CDH1* gene deletion (Figure 1). Since both *CDH1* gene mutation and deletion lead to an abnormal or loss of E-cadherin function, we combined *CDH1* mutations and deletions into a *CDH1* “altered” group in this analysis. Together, 59.2% of the TCGA ILC tumors (100/169) had a *CDH1* alteration and the remaining 40.8% (69/169) had a normal *CDH1* genetic status. In contrast, IDC and ICDL had a significantly lower prevalence of *CDH1* gene alterations (2.6%, 18/690 and 16.7%, 6/36, respectively).

Surprisingly, no significant difference was identified between the “*CDH1*-altered” and “*CDH1*-unaltered” ILC groups in any of the demographics or clinical or pathological characteristics, including tumor size, histologic grade, lymph node metastasis, stage, or hormonal receptor (ER/PR/HER2) states (Table 1). While it has been well recognized that an ILC diagnosis should be made histologically regardless of its E-cadherin expressional status as determined by immunostaining [7], our data further suggests that a diagnosis of ILC also should not be affected by its status of a *CDH1* gene alteration, since no difference was identified in the pathological characteristics, nor in the prognosis between the ILC with or without a *CDH1* alteration. These results also make the TCGA ILC cohort an ideal case series with minimal confounding bias for us to further compare the molecular differences between these two groups.

**Table 1:** Clinical and pathological information of TCGA ILC cohort with (*CDH1*+) and without (*CDH1*-) *CDH1* alteration

Categories	Categories	*CDH1*+ (*n* = 100)		*CDH1*− (*n* = 69)		*p* value
		Cases	%	Cases	%	
Ethnicity	Asian	4	4.0	3	4.3	0.91
	Black	7	7.0	3	4.3	0.47
	White	79	79.0	58	84.1	0.41
	Unknown	10	10.0	5	7.2	
Menopause Status	Peri	2	2.0	0	0.0	0.24
	Post	77	77.0	51	73.9	0.65
	Pre	14	14.0	13	18.8	0.40
	Unknown	7	7.0	5	7.2	
Tumor size	T1	13	13.0	12	17.4	0.43
	T2	54	54.0	39	56.5	0.75
	T3	32	32.0	18	26.1	0.41
	T4	1	1.0	0	0.0	0.40
Lymph node status	N0	49	49.0	30	43.5	0.48
	N1	27	27.0	21	30.4	0.63
	N2	8	8.0	6	8.7	0.87
	N3	16	16.0	12	17.4	0.81
Metastasis	M0	75	75.0	54	78.3	0.30
	MX	25	25.0	15	21.7	0.62
Stage	Stage I	7	7.0	8	11.6	0.30
	Stage II	59	59.0	39	56.5	0.75
	Stage III	32	32.0	22	31.9	0.99
	Unknown	2	2.0	0	0.0	
Surgical procedure	Lumpectomy	18	18.0	13	18.8	0.89
	Modified Mastectomy	31	31.0	22	31.9	0.90
	Simple Mastectomy	28	28.0	23	33.3	0.46
	Other	23	23.0	9	13.0	0.10
	Unknown	0	0.0	2	2.9	
Postop radiotherapy	NO	9	9.0	8	11.6	0.58
	YES	11	11.0	9	13.0	0.69
	Unknown	80	80.0	52	75.4	
ER	Negative	4	4.0	3	4.3	0.91
	Positive	94	94.0	64	92.8	0.75
	Unknown	2	2.0	2	2.9	
PR	Negative	17	17.0	7	10.1	0.21
	Positive	81	81.0	59	85.5	0.44
	Unknown	2	2.0	3	4.0	
HER2	Equivocal	6	6.0	3	4.3	0.64
	Negative	78	78.0	52	75.4	0.69
	Positive	10	10.0	10	14.5	0.37
	Unknown	6	6.0	4	5.8	

### Genomic changes associated with a *CDH1* gene alteration in ILC

**Figure 2:**
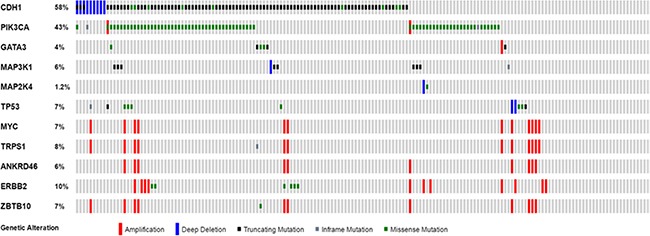
Distribution of cancer driver genes in ILC with and without a *CDH1* alteration The data is from Breast Invasive Carcinoma, TCGA provisional, and plotted using Oncoprint from the cBioportal. No statistical difference (Fisher's exact test, *p* > 0.05) was identified in all 10 cancer driver genomic abnormalities as defined in our previous study (12) between the ILC with and without a *CDH1* alteration.

Although rare high grade ILC variants exist, such as pleomorphic invasive lobular carcinoma (pILC), ILC is generally a histologically low grade but clinically intermediate grade invasive breast cancer [1, 2]. This is also supported by our previous TCGA breast cancer genomic study demonstrating that ILC carries a moderate mutational burden as compared to IDC and other histologic variants of invasive breast cancer [13]. Among the known breast cancer driver genes, *PIK3CA* is the most frequently mutated gene associated with a low histologic grade in IDC [12]. In ILC, a *PIK3CA* mutation is the second most common genomic abnormality identified (43.2%, 73/169, Figure 2), only next to the ILC hallmark *CDH1* gene mutation. Interestingly, although both are associated with low grade tumors, the presence of a *PIK3CA* mutation is not associated with a *CDH1* mutation and occurs in a similarly high prevalence in ILC with and without a *CDH1* mutation (Figure 2). Two well-known breast cancer driver genomic abnormalities, *TP53* mutation and *MYC* amplification, which are typically seen in high grade IDC, are seen in a much lower incidence in ILC than in IDC, with a pattern similar to the low to intermediate grade IDC [12]. Both *TP53* mutation and *MYC* amplification are also not correlated with a *CDH1* mutation (Figure 2). These data suggests that the ILC with and without a *CDH1* alteration share a common oncogenic mechanism similar to low to intermediate grade IDC during cancer initiation and progression.

**Figure 3:**
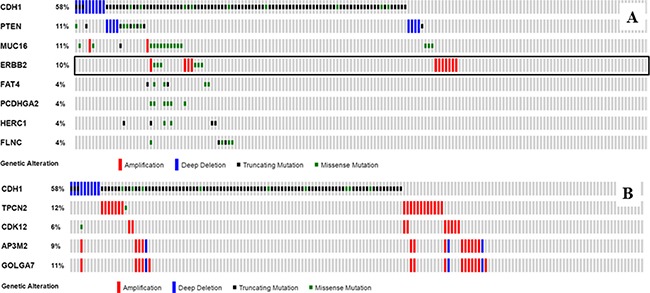
Significant mutations and CNVs associated with *CDH1* alterations in ILC The data is from Breast Invasive Carcinoma, TCGA provisional, and plotted using Oncoprint in the cBioportal. Seven recurrent mutations and four chromosomal loci with recurrent CNV were identified with a significant difference (Fisher's exact test, *p* < 0.05) between the ILC with and without a *CDH1* mutation. *TPCN2*, *CDK12*, *AP3M2* and *GOLGA7* are representative gene amplifications in chromosomal regions 11q13, 17q12-21, 8p11 and 8q11, respectively. The black frame highlights a significant difference in ERBB2 gene mutation, but no significant difference in *ERBB2* gene amplification between the ILC with and without a *CDH1* mutation. (**A**) significant mutation; (**B**) significant CNV.

While there is no significant clinical or pathological difference between the ILC with and without a *CDH1* alteration (Table 1), the number of significant genomic changes associated with a *CDH1* gene alteration appears to be also limited. Among 47 genes with recurrent mutations and 176 chromosomal loci with recurrent gene amplifications in a prevalence ≥ 2%, seven recurrent gene mutations involving *PTEN, MUC16, ERBB2, FAT4, PCDHGA2, HERC1* and *FLNC* genes (Figure 3A) and four chromosomal loci with recurrent copy number variation (11q13, 17q12-21, 8p11 and 8q11, Figure 3B) were identified to be significantly different between the two groups. Interestingly, these seven significant mutations were mostly observed in ILC with a *CDH1* alteration, in contrast, all the significant CNVs were more often seen in the ILC without a *CDH1* alteration.

### Prognostic significance of a *CDH1* alteration and associated genetic changes

**Figure 4:**
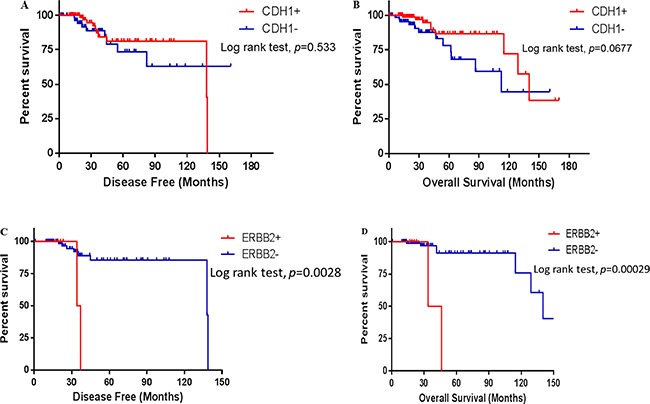
Survival of the ILC patients in different states of *CDH1* and *ERBB2* gene mutation states The survival data is from Breast Invasive Carcinoma, TCGA provisional, and the survival difference was analyzed and plotted using Graphpad Prism 6.0. A Log-rank test was used to examine the statistical difference between the ILC patients with (*CDH1*+) and without (*CDH1*−) a *CDH1* gene mutation, and between the *CDH1*-altered ILC patients with (*ERBB2*+) and without (*ERBB2*−) an *ERBB2* gene mutation. *p* < 0.05 is considered as statistically significant. (**A, C**) disease-free survival; (**B, D**) overall survival.

Loss of E-cadherin is the predominant and hallmark genetic abnormality in ILC, however, a *CDH1* gene alteration alone was not associated with altered survival in the patients with ILC (Figure 4A and 4B). Among those significant mutations and CNVs associated with a *CDH1* alteration, *ERBB2* was the most well-known oncogene associated with high grade breast cancer [14]. However, no significant difference was observed in the incidence of *ERBB2* amplification between the ILC with and without a *CDH1* alteration (Figure 3). In contrast, all *ERBB2* mutations were identified in the ILC with a *CDH1* alteration. While *ERBB2* amplification is not associated with prognosis in ILC patients irrespective of *CDH1* alteration status, the *CDH1*-altered ILC patients with an *ERBB2* mutation showed a significantly worse prognosis as compared to their counterparts without an *ERBB2* mutation (Figure 4C and 4D). No other recurrent mutation was found to have a significant effect on either overall or disease-free survival in the *CDH1*-altered ILC, including the *PTEN* mutation, which was recently reported to be associated with AKT pathway activation in ILC [9]. For those known breast cancer driver genes such as mutations in *TP53, PIK3CA, FOXA1* and amplifications in *MYC, ERBB2* and *CCND1*, again no survival difference was observed to be associated with the presence of such genetic abnormalities.

**Figure 5:**
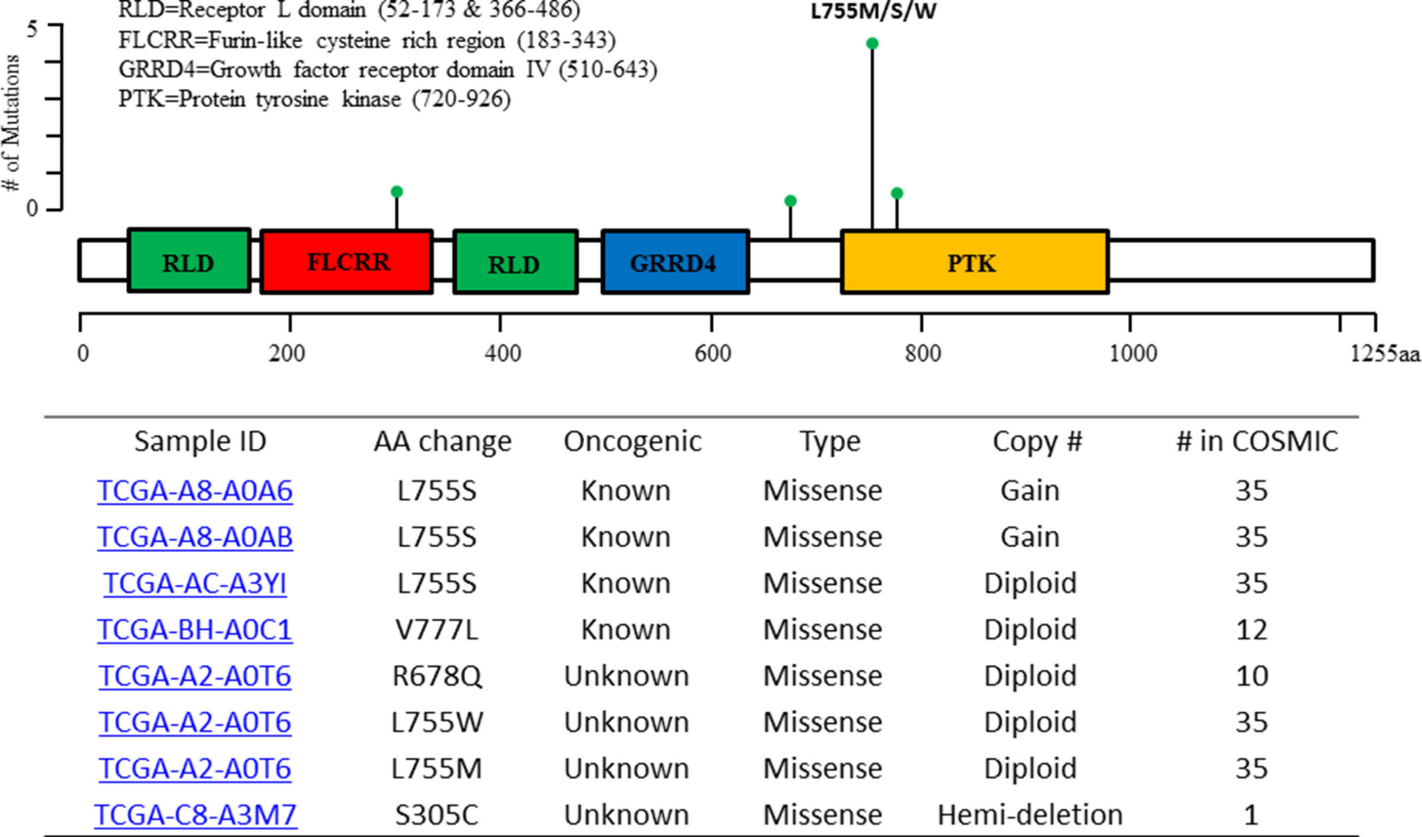
Distribution of *ERBB2* gene mutations in the patients with *CDH1*-altered ILC aa: amino acid; COSMIC: the Catalogue Of Somatic Mutations In Cancer.

*ERBB2* mutations were found in six of the 100 *CDH1*-altered ILC (Figure 3). The vast majority of these tumors (5/6) carried mutations in the tyrosine kinase domain of the *ERBB2* protein. L755S was the most frequent recurrent mutation (Figure 5), and was identified in half of the cases (3/6). Four of these mutations were known to be oncogenic and could be treated by Neratinib, a second-generation HER2/EGFR tyrosine kinase inhibitor [15]. Notably, one tumor carried three mutations, R678Q, L755W and L755M. One rare mutation in the *ERBB2* furin-like domain, S305C, was also identified, which was reported only once in the COSMIC database. Interestingly, in contrast, no *ERBB2* mutation was found in *CDH1*-unaltered ILC (0/69).

## DISCUSSION

Invasive lobular carcinoma is a well-characterized histologic variant and the second most common histological subtype of invasive breast cancer. It is molecularly distinct from other subtypes of breast cancer featured by aberrant or loss of E-cadherin protein expression [1, 2]. However, according to recent genome sequencing studies, only 30–60% of the ILC tumors carry a *CDH1* gene mutation or deletion (COSMIC data, 9–11). The remaining subset of ILC, although displaying a lobular histologic phenotype, may be caused by other molecular mechanisms that lead to a common end point of cell adhesion abnormalities that are associated with a dysfunctional or lost E-cadherin protein [9]. The causes could be an abnormal or lost *CDH1* gene or protein expression as well as abnormalities of other components of the E-cadherin signaling pathway, such as α, β, γ and p120 catenins [2]. In this study, we compared the clinical, pathological and genomic differences of ILC with and without a *CDH1* alteration in 169 cases of ILC patients from TCGA. Surprisingly, no significant difference was identified in any of clinical or pathological features between the ILC with and without a *CDH1* alteration. These findings were validated by analyzing the data from another large cohort ILC genomic study [10]. All the driver genomic abnormalities identified in IDC [12], including mutations in *TP53* and *PIK3CA* genes and amplifications in *MYC* and *ERBB2*, are not associated with *CDH1* alteration status. These findings support the concept that ILC is not only a histologically unique variant of invasive breast cancer, but also a molecularly distinct entity characterized by a lost or dysfunctional E-cadherin protein [2]. This view is also supported by a recent TCGA ILC study which revealed that up to 95% of TCGA ILC cases carry genetic changes in the *CDH1* gene, including somatic mutations, copy-number losses, and alterations in mRNA and protein expression [9]. In this case, most TCGA ILC will display negative or decreased E-cadherin protein expression by IHC, which is similar to the reported rate of E-cadherin protein loss (84%) as measured by IHC [7]. While it has been well recognized that a diagnosis of typical ILC should be made on the basis of its histology and is not necessary be confirmed by E-cadherin immunostaining, our study further importantly suggests that a histologic diagnosis of ILC should not be affected by its mutational status of the *CDH1* gene.

Interestingly, our study has shown that the most significant recurrent gene mutations that correlated with *CDH1* mutational status occurred only in those ILC with a *CDH1* alteration, and are absent in the ILC without a *CDH1* alteration. In contrast, the most recurrent significant CNVs that correlated with *CDH1* mutational status were often identified in the ILC without a *CDH1* alteration, but are rare in the ILC with a *CDH1* alteration. This unique genomic feature suggests that the histologically uniform ILC is, in fact, molecularly heterogeneous and could be further classified into at least two main subtypes by its state of *CDH1* alteration. This finding is in line with a recent pan-cancer genomic analysis regarding global molecular classification across a diverse group of 12 human cancer types. This study revealed two top tumor classes with distinct types of genomic aberrations at the first partition of the pan-cancer data analysis by *hierarchical clustering*, the mutation (M) class and the altered copy number (C) class. They exist in an inverse relationship and represent two different levels of the oncogenic processes [16]. Similar mutation versus amplification distribution disparity was found to be correlated to low and high histologic grade, respectively, and was also reported in IDC of the breast recently by us [12]. In ILC, a *CDH1* mutation was found to be clustered with a group of significant gene mutations and appears to be mutually exclusive to any significant CNV. However, this unique genomic pattern is not associated with any common pathological or prognostic features, including histologic grade, and its significance remains unknown. Our data suggests that although a *CDH1* alteration is the diagnostic hallmark of ILC and is the genetic basis of the lobular phenotype of most ILC, a *CDH1* alteration itself (and its associated genomic changes) may not be as important in determining the prognosis of this most common non-ductal variant of breast cancer.

Our study has also revealed that those most common breast cancer driver abnormalities, i.e. *TP53* and *PIK3CA* mutations and *MYC* and *ERBB2* amplifications are not associated with *CDH1* gene alteration status. A *PTEN* mutation and a *FOXA1* mutation were recently found to be associated with activation of the AKT pathway and increased ER activity, respectively, in ILC [9]. Annunziato et al. have also showed that disruption of *PTEN* in the mouse mammary gland lacking E-cadherin expression using CRISPR/Cas9 gene editing lead to efficient development of ILC in the mouse model [17]. In TCGA ILC cohort, *PTEN* mutation occurs more frequently in *CDH1*-altered ILC (6 of 100 or 6% vs 0/69, respectively, *p* < 0.05). A similar but statistically insignificant trend (12/270 or 4.4% vs 4/143 or 2.8%) was also observed in another large ILC sequencing study [10]. However, both *PTEN* and *FOXA1* mutational states, as well as CCND1 amplification/overexpression states that are frequently observed in ER-positive breast cancer had no significant effect on overall or disease free survival in relation to the *CDH1* alteration status in TCGA ILC cohort.

*ERBB2* amplification is a known oncologic driver in breast cancer, however, the role of *ERBB2* mutation is not well defined [18]. While no synergistic effect is observed between *CDH1* mutation and *ERBB2* amplification, the *CDH1*-altered ILC with an *ERBB2* mutation shows a significantly worse prognosis compared to its *CDH1*-unaltered counterparts. Our observation is in line with a recent genomic study which showed that the relapsed classic E-cadherin mutated ILC had a high frequency of *ERBB2* gene mutation (4/22, 18%) [19]. *CDH1* mutations were also found enriched in *ERBB2* mutated metastatic breast cancer and associated with its recurrence [20]. Significantly, our finding has been further validated using the data from another large cohort of ILC sequencing study, in which 18 *ERBB2* mutations were found in 270 cases of ILC with a *CDH1* alteration while only three *ERBB2* mutations were present in their *CDH1*-unaltered counterparts [10]. Interestingly, in this study, a survival analysis shows that the *CDH1*-mutated ILC patients with an *ERBB2* mutation had a worse 5-year survival (*p* < 0.05), but a similar 10 years survival as compared to their counterparts without an *ERBB2* mutation (*p* > 0.05, data not shown). While the causes of this controversy could be multifactorial, including limited cases studied and heterogeneity in tumor genetics and patient treatment, our finding is clinically significant since most *ERBB2* mutations are known to be oncogenic [18] and were identified in a significantly high percentage of recurrent ILC patients [19]. Importantly, cancers with *ERBB2* mutations could be treated by Neratinib, an irreversible pan-HER tyrosine kinase inhibitor, which is currently in late-phase clinical development. Therefore, *ERBB2* mutation is an actionable genetic abnormality and identification of *ERBB2* mutation in *CDH1*-altered ILC patients may be an important step towards targeted therapy of this group of ILC patients [15, 21–23].

In summary, our study supports ILC as a morphologically and molecularly distinct variant of invasive breast cancer. Furthermore, ILC patients with tumors carrying both *CDH1* and *ERBB2* mutations have a worse prognosis, but represent an actionable group who may benefit from targeted breast cancer therapy.

## MATERIALS AND METHODS

### TCGA invasive lobular carcinoma data

The Breast Invasive Carcinoma (TCGA, Provisional) dataset was accessed as described previously [12, 13]. This data set, as of July 1, 2016, contains the genomic sequencing data encompassing gene mutations, copy number alterations (CNA), mRNA and protein expression as well as clinical and pathological data including patient survival, pathology reports and digitized tumor slides from 1105 samples from 1098 patients with invasive breast cancer. The cohort consists of 690 IDC cases, 169 ILC cases, 36 invasive carcinoma with ductal and lobular features (ICDL) cases and 210 cases of other subtypes of invasive breast cancer. All TCGA data were accessed and analyzed using cBioPortal (http://www.cbioportal.org/) as described previously [12, 13].

### Statistical analysis

All the results for continuous variables are presented as the mean ± SD, and the results for the categorical variables are presented as the number of cases and percentage. The significance of the differences between the groups was assessed using Student's *t-test* for the continuous variables and the Fisher's exact test for the categorical variables. All tests were 2-tailed, and a *p-value* of 0.05 was considered to be statistically significant. The survival differences were analyzed by a Log-rank test using the GraphPad Prism 6 software (GraphPad Software, Inc, La Jolla, CA). The data were analyzed with XLSTAT for Windows, version 2016.02 (Addinsoft, New York, NY).
